# Selenium Enhances Osteogenic Differentiation and Mineralization in Human Osteoblasts: Implications for Bone Health and Metabolism

**DOI:** 10.2174/0109298673391434250430053916

**Published:** 2025-08-21

**Authors:** Erhan Sahin, Mahmoud Arafat, Ayse Tansu Koparal

**Affiliations:** 1 Histology and Embryology Department, Faculty of Medicine, Bilecik Seyh Edebali University, Bilecik, Türkiye;; 2 Department of Medical Services and Techniques, Yunus Emre Vocational School of Health Services, Anadolu University, Eskisehir, Türkiye

**Keywords:** hFOB 1.19 cells, sodium selenite, osteogenic differentiation, osteoblastic activity, ALP enzyme, selenium

## Abstract

**Introduction:**

Sodium Selenite (NaSe) is a molecule with various biological activities. Bone fractures and osteoporotic diseases are increasingly common health issues, prompting the search for alternative treatments. Therefore, the purpose of this study was to examine the antioxidant and osteogenic properties of NaSe.

**Methods:**

The experiments were conducted using the hFOB1.19 osteoblast cell line. The MTT assay was used to assess the effects of NaSe on cell viability, while cytotoxicity was evaluated with Lactate Dehydrogenase (LDH) assays. Osteogenic differentiation was assessed by alizarin red staining, and Alkaline Phosphatase (ALP) activity and intracellular Reactive Oxygen Species (ROS) levels were also analyzed.

**Results:**

The results showed that NaSe significantly enhanced cell viability in a dose-dependent manner at low doses (0.01-1μM), with the most effective dose being 1μM (*p*<0.05). LDH activity remained similar to the control within the 0.01-1μM range but increased significantly at higher concentrations (5-50 μM) in both 24- and 48-hour experiments (*p*<0.05). NaSe reduced intracellular ROS levels significantly between 0.01-1 μM, with 1 μM being the most effective concentration (*p*<0.05). The highest ALP activity was observed at 0.1 μM NaSe (*p* < 0.05), and calcium deposition increased in a concentration-dependent manner (*p*<0.05). The most effective dose for enhancing mineralization was 0.1 μM (*p*<0.05).

**Conclusion:**

This study demonstrates that NaSe has antioxidant and osteogenic effects at low doses in hFOB cells. These positive effects suggest that NaSe could be a promising candidate for *in vitro, in vivo*, and clinical trials, providing hope for new treatments for bone diseases.

## 1 INTRODUCTION

Ossification, the process by which bone tissue is formed, is a complex and tightly regulated biological phenomenon crucial for skeletal development and maintenance [1]. Two primary types of ossification exist: intramembranous and endochondral ossification. Intramembranous ossification involves the direct formation of bone within a connective tissue membrane, while endochondral ossification occurs within a cartilage template. These processes are orchestrated by a multitude of signaling pathways, growth factors, trace elements, and transcription factors that govern the differentiation and maturation of osteoblasts, the bone- forming cells. Understanding the molecular mechanisms underlying ossification is essential for insights into skeletal disorders and therapeutic interventions [2-4]. Enhancing osteoblastic activity could offer hope for the treatment of various bone diseases. The intake of various trace elements from external sources plays a substantial role in influencing bone metabolism within our bodies. One of these trace elements is selenium.

Sodium selenite (NaSe), a chemical compound with the formula Na_2_SeO_3_, serves as a source of selenium, an essential trace element vital for various biological processes. Selenium is known for its antioxidant properties and its involvement in the synthesis of selenoproteins, which are critical for cellular function and protection against oxidative stress. NaSe has been explored for its potential therapeutic benefits, especially in cancer prevention and treatment, owing to its ability to trigger apoptosis in cancer cells [5]. The comprehensive exploration of NaSe's biological activities underscores its significance in health and disease [6-8]. Evidence highlights the essential role of selenium in maintaining healthy bone metabolism, underscoring the importance of sufficient selenium intake for skeletal health [9, 10]. However, a detailed study of the osteoblastic activity of NaSe under *in vitro* conditions will serve as the basis for *in vivo* and clinical studies targeting NaSe and ossification.

In the framework of our study, our aim is to explore the impact of NaSe on the osteogenic functions of hFOB 1.19 human osteoblast cells in an *in vitro* environment.

## 2 MATERIALS AND METHODS

### 2.1 Cell Culture

hFOB 1.19 cells (ATCC, Manassas, VA, USA, CRL-11372) were grown in 75 cm^2^ flasks using a phenol red-free DMEM: Ham's F12 (1:1) medium supplemented with 10% fetal bovine serum. Phenol red-free media were used in cell culture in hormone-sensitive studies to reduce background noise, prevent pH-related artifacts, and ensure compatibility with sensitive biochemical tests. It is essential for endocrine research, stem cell cultures, and high-throughput screening but unnecessary for routine cell maintenance. The media also included 0.3 mg/ml geneticin (Gibco, G418) and 2.5 mM L-glutamine. The cells were incubated in a 5% CO_2_ atmosphere at 34°C. Experiments were initiated once the cells achieved 70% confluency in the flasks.

### 2.2 Preparation of NaSe Doses

To prepare NaSe solutions, a stock solution was made by dissolving NaSe (Sigma) in Phosphate-Buffered Saline (PBS). Concentrations ranging from 0.01 to 50 μM (0.01, 0.05, 0.1, 1, 5, 10, 25, and 50 μM) were prepared by diluting the stock solution with PBS. PBS alone served as the negative control, as NaSe was dissolved in PBS. The NaSe stock solution was stored at -20°C for future use.

### 2.3 Viability Assays

The effect of NaSe on cell viability was evaluated using the MTT [3-(4,5-dimethylthiazol-2-yl)- 2,5-diphenyltetrazolium bromide] assay. In the MTT experiments, NaSe concentrations tested ranged from 0.01 to 50 μM (0.01, 0.05, 0.1, 1, 5, 10, 25, and 50 μM). Cells were grown in incubators with 95% air and 5% CO_2_ at 34°C until they reached 70% confluency. Once 70% confluency was achieved, cells were detached from the flask surface using trypsin-EDTA solution (which includes tetrasodium ethylenediaminetetraacetate dihydrate) to create a cell suspension.

Cell counting was conducted three times using a Thoma chamber, and 5,000 cells were seeded into each well of 96-well culture plates. After a 24-hour incubation to allow cell attachment, the media were discarded by inverting the plates. Cells were then treated with varying concentrations of NaSe for 24 and 48 hours. Post-treatment, the NaSe-containing media were removed, and 0.5 mg/ml MTT solution was added to each well for a 2-hour incubation. Subsequently, the MTT solution was replaced with 0.1 ml of dimethyl sulfoxide (DMSO). The absorbance of the wells was measured at 570 nm using an ELISA reader. The viability of the untreated control group was defined as 100%, and the viability of treated cells was calculated as a percentage relative to the control. To reduce variability, each NaSe concentration was applied to 8 individual wells in the 96-well plate, and the results from these wells were averaged for each concentration. The MTT assay was repeated three times to ensure the reliability of the findings [11].

### 2.4 Cytotoxicity Assays (Lactate Dehydrogenase Release Assay)

The Lactate Dehydrogenase (LDH) cytotoxicity detection kit (Roche, Germany, Cat. No. 04744926001) was used to perform the LDH release assay. In a 96-well cell culture plate, 5,000 cells were seeded per well. The cells were allowed to attach to the well surface during a 24-hour incubation period. After incubation, the media were removed by inverting the plates. The cells were then treated with different concentrations of NaSe for 24 and 48 hours. Following NaSe exposure, the supernatant was collected, and the LDH assay was carried out according to the manufacturer’s protocol. The optical density of the plates was measured at 492 nm using a microplate reader to quantify the amount of LDH enzyme released.

### 2.5 Measurement of Intracellular Reactive Oxygen Species Levels

The Biovision kit (K936-250, Milpitas, CA, USA) was used to measure intracellular Reactive Oxygen Species (ROS) levels. Cells were plated in a 96-well plate at a density of 5,000 cells per well. After allowing 24 hours for cell attachment, the medium was discarded, and 100 µl of ROS assay buffer was added to each well as per the kit’s instructions. The cells were washed, and 100 μL of 1X ROS marker, diluted in ROS assay buffer, was introduced into the wells. The plate was then incubated at 37°C for 40 minutes. After incubation, the 1X ROS marker was removed, and freshly prepared NaSe concentrations (0.01, 0.05, 0.1, 1, 5, 10, and 25 μM) were added to the wells. The plates were incubated again at 37°C for 24 hours. Following this incubation, changes in intracellular ROS levels were quantified by measuring fluorescence at 495/529 nm. The results were analyzed by comparing them to the control group.

### 2.6 Facilitating the Osteogenic Differentiation Process in hFOBs

To promote differentiation in cultured cells, an osteogenic differentiation medium (OD (+)) was added. The OD (+) medium was composed of 0.3 mg/ml geneticin (Gibco, G418), 0.05 mM ascorbic acid 2-phosphate, 10 mM β-glycerophosphate, 2.5 mM L-glutamine, 10^−8^ M dexamethasone, and phenol red-free DMEM: Ham's F12 (1:1) supplemented with 10% fetal bovine serum. To maintain the osteogenic differentiation process, the OD (+) medium was replaced every 3-4 days.

Samples were collected for further biochemical analysis (on days 7 and 14). In comparison, the medium without the osteogenic differentiation medium (OD (-)) contained 2.5 mM L-glutamine, 0.3 mg/ml geneticin (Gibco, G418), and phenol red-free DMEM: Ham's F12 (1:1) with 10% fetal bovine serum.

### 2.7 Assessment of Osteogenic Differentiation Using Alkaline Phosphatase Activity

The biochemical evaluation of osteogenic differentiation in cultured cells included measuring the activity of the Alkaline Phosphatase (ALP) enzyme. NaSe concentrations of 0.01, 0.05, 0.1, and 1 µM were tested in the ALP assays. Cells were plated in 24-well plates at a density of 4×10^4^ cells/well using both OD (+) and OD (-) media. On days 7 and 14, the media were removed from the incubated cells, and the cells were washed once with PBS before being stored at -80°C. After thawing on ice for 20 minutes, a lysis buffer (PBS containing 1% Triton-X-100) was added to each well. The plates were sonicated in an ice bath for about 50 minutes, and the lysates were transferred to tubes using a pipette. For each sample, 20 μl of the cell lysate was transferred to 96-well plates. A 100 μl ALP substrate solution (prepared by dissolving 1 p-nitrophenylphosphate tablet in 20 ml of ALP substrate buffer [0.1 M diethanolamine, 1% Triton-X-100, 1 mM MgCl_2_, pH 9.^8^]) was added to each well. The mixture was briefly shaken and incubated at 37°C for 30 minutes to allow the ALP enzyme to convert pNpp into paranitrophenol. The experiment was repeated three times, with at least two wells used for each sample. After 30 minutes, the reaction was stopped by adding 80 μl of 1N NaOH, and absorbance was measured at 405 nm using an ELISA reader.

### 2.8 Assessment of Osteogenic Differentiation using Alizarin Red Staining

The calcium deposition activity of hFOB cells was visualized microscopically using the Alizarin Red (AR) staining technique. In the AR experiments, NaSe concentrations of 0.01, 0.05, 0.1, and 1 µM were tested. After 28 days, the medium was carefully aspirated from the cells in the 24-well plate. The plates were then fixed with 70% cold ethanol at room temperature for 1 hour. Following fixation, the ethanol was removed, and the plates were rinsed twice with distilled water for 5 minutes each. The water was then discarded, and 0.1% AR solution was added to the wells, followed by a 30-minute incubation. After removing the AR solution, the plates were washed four times with distilled water. The stained cells were observed under a microscope, and images were captured to assess calcium deposition. The experiment was repeated three times to ensure consistent and reliable results.

### 2.9 Image Processing and Analysis

Images captured under the microscope were saved in JPEG format. For quantitative analysis, the ImageJ software was utilized. First, the selected image was opened in ImageJ. The images were then converted to 8-bit format using the 'Image > Type > 8-bit' option in the program menu. To define the parameters for analysis, the 'Area' and 'Area Fraction' options were selected under 'Analyze > Set Measurements'. Next, threshold values for the images were established using the 'Image > Adjust > Threshold' function. The results for each image were generated by selecting 'Analyze > Measure'. The data obtained were exported to Excel for subsequent statistical evaluation.

### 2.10 Statistical Analysis

All experiments were performed in triplicate, and the data were analyzed using statistical software. Statistical significance was set at *p*<0.05. The Shapiro-Wilk test was used to evaluate the normal distribution of the data. For data following a normal distribution, one-way ANOVA was conducted, followed by Tukey’s post-hoc test for multiple comparisons.

## 3 RESULTS

### 3.1 Viability Results

**Fig. (1):**
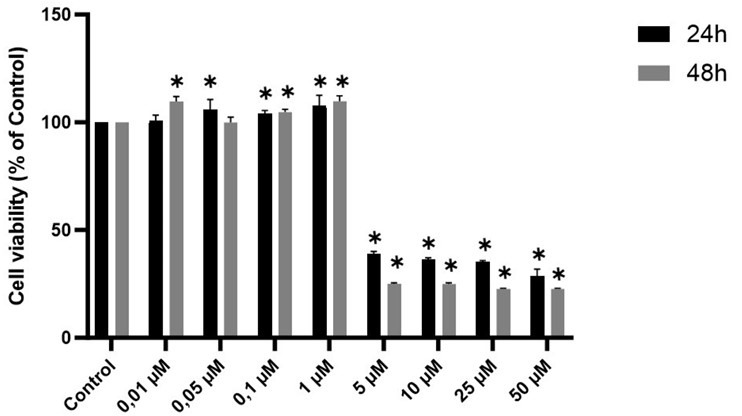
MTT Results. NaSe enhanced cell viability up to 1 µM at both 24 and 48 hours, but viability declined sharply at concentrations above 1 µM. *Statistically significant compared to the untreated control group (*p* < 0.05).

The effects of NaSe on hFOB cells were examined over 24 and 48 hours. At low concentrations of up to 1 μM, NaSe significantly enhanced cell viability in a dose-dependent manner (*p*<0.05). The most effective dose that increased cell viability in both application periods was found to be 1μM. Cell viability increased by approximately 10% in 1 μM administered groups. NaSe at doses between 5 and 50 μM meaningfully reduced cell viability (*p*<0,05). In groups administered with 50 μM, the viability of the cells decreased by 72% in 24 hours, while the viability of the cells decreased by 78% in 48 hours (Fig. **1**).

### 3.2 Cytotoxicity Results

**Fig. (2):**
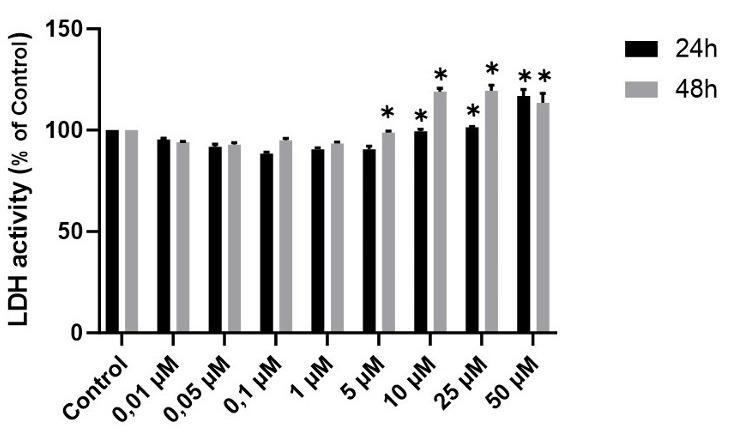
LDH Results. LDH activity remained similar to the control group between 0.01-1 µM. However, a significant increase in LDH activity was observed at concentrations between 5 and 50 µM in both 24- and 48-hour experiments (*p*<0.05). *Statistically significant compared to the untreated control group (*p*<0.05).

In our experiment, LDH activity determination was performed to determine cellular damage. The kit measured LDH activity released into the supernatant from the cytosol of injured cells. Between 0.01-1 μM, LDH activity was similar to control. It was determined that LDH activity increased significantly between 5 and 50 μM in 24- and 48-hour experiments (*p*<0.05, Fig. **2**).

### 3.3 ROS Results

**Fig. (3):**
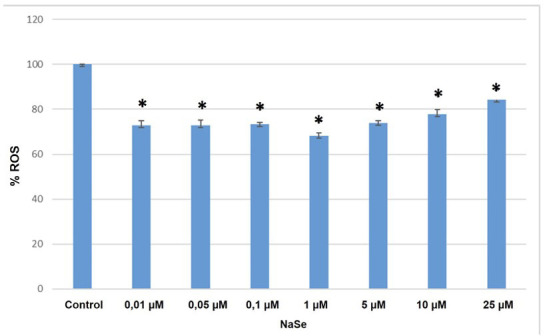
ROS Scavenging Results. NaSe significantly reduced intracellular ROS levels between 0.01-1 µM (*p* < 0.05), with the most effective concentration being 1 µM. Above 5 µM, ROS levels increased in a concentration-dependent manner (*p* < 0.05). *Statistically significant compared to the untreated control group (*p*<0.05).

The ROS Detection Assay Kit is intended for identifying hydroxyl, peroxyl, and other reactive oxygen species within living cells. In our experiment, it was determined that NaSe significantly reduced the amount of intracellular ROS between 0.01-1 μM (*p*<0.05), and the most effective concentration was 1 μM. After 5 μM, the amount of ROS increased in parallel with the rising concentration (*p*<0.05, Fig. **3**).

### 3.4 ALP Enzyme Results

**Fig. (4):**
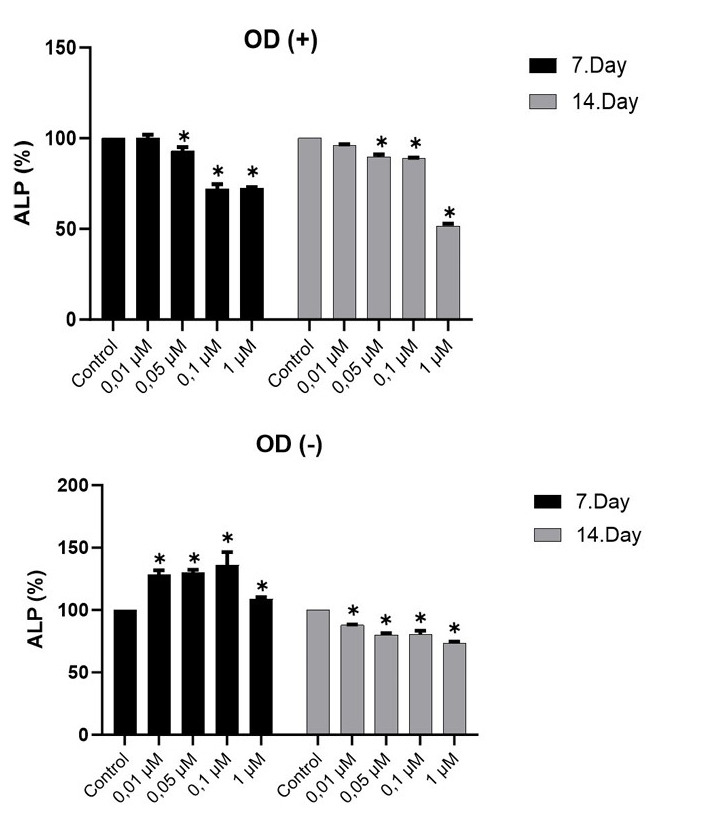
ALP Enzyme Results. In the OD (-) group, a statistically significant increase in ALP enzyme was observed only at the 7-day time point (*p*<0.05). The highest ALP enzyme level was detected at a NaSe concentration of 0.1 µM (*p*<0.05). In other treatments, ALP activity decreased compared to the control. *Statistically significant compared to the untreated control group (*p*<0.05).

The biochemical assessment of osteogenic differentiation in cultured cells was performed by quantifying ALP enzyme activity on days 7 and 14. Various NaSe concentrations (0.01, 0.05, 0.1, and 1 µM) were tested in both experimental setups with osteogenic differentiation medium (OD (+)) and without osteogenic differentiation medium (OD (-)). In the OD (-) group, a statistically significant rise in the ALP enzyme was exclusively noted in the 7-day timeframe (*p*<0.05). The ALP enzyme exhibited its highest detection level at a NaSe concentration of 0.1 µM (*p*<0.05). No significant alteration in ALP enzyme levels was detected in any of the other treatment groups (Fig. **4**).

### 3.5 Alizarin Red Dye Results

**Fig. (5):**
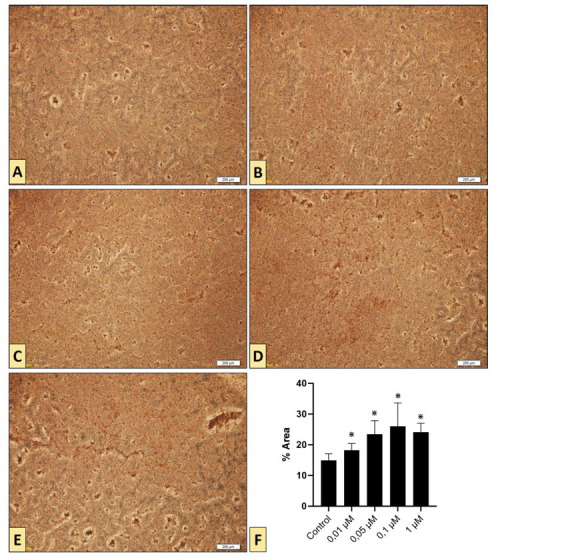
Calcium Deposit Results (Alizarin Red Staining). (**A**-**F**) Respectively Control, 0.01 µM, 0.05 µM, 0.1 µM, 1 µM NaSe-treated cells and image analysis results of sections stained with Alizarin Red. Calcium accumulation rose in a dose-dependent fashion (*p*<0.05). The most effective dose-promoting mineralization in hFOB cells was 0.1 µM (*p* < 0.05). The parts of the figures colored red represent calcium deposits. *Statistically significant compared to the untreated control group (*p*<0.05). All scale bars represent 200 µm.

To demonstrate the osteogenic activity of NaSe in hFOB cells, we cultured the cells in various concentrations of NaSe for 28 days and performed AR staining to observe calcium deposits of hFOB cells at the end of the experiment. It was found that calcium accumulation rose in a dose-dependent manner (*p*<0.05). The most important dose that positively affected mineralization in hFOB cells was determined to be 0.1µM (*p*<0.05, Figs. **5A**-**F**).

## 4 DISCUSSION

Selenium is a trace element that contributes to very important physiological processes within the cell. In this study, we investigated the effects of NaSe, a source of selenium, on hFOB cells, which are osteoblast cells. To the best of our knowledge, this is the first study to investigate the effects of NaSe on hFOB cells, making its findings novel and significant. We found that NaSe had an osteoblastic and antioxidant effect on hFOB cells at low doses. We determined that 0.1 µM NaSe had the highest osteoblastic activity in hFOB cells.

Beyond conventional therapies for bone diseases, growing research highlights the potential of natural alternative agents, among which selenium [12] emerges as a promising candidate due to its potent biological activity. Selenium deficiency in humans can lead to serious metabolic disorders [13].

Therefore, it is very important to have enough selenium in our diet. Cereals, meat, fish, and eggs are foods rich in selenium. Selenium is converted into selenoproteins within the body. These selenoproteins are the structures that provide antioxidant and other beneficial physiological activities of selenium [14-18].

In our experiment, NaSe enhanced cell viability in osteoblast cells up to 1 µM, but significantly reduced viability by more than 50% at concentrations above 1 µM. This highlights the effectiveness of low NaSe doses. The optimal 1 µM dose demonstrates selenium’s biphasic effect at low concentrations, and it may function as a micronutrient, boosting cell viability by supporting antioxidant defenses (*e.g.*, selenoproteins), while higher doses (≥5 µM) induce oxidative stress and cytotoxicity.

Comparing our study with that of Liu *et al.*, we observe that hFOB cell viability drops below 50% at 5 µM after 24 hours, whereas kidney cancer cells maintain around 60% viability at the same dose [19]. This suggests that cancerous cells may be more resistant to NaSe. These findings highlight the importance of considering cancer cell characteristics when determining dosing in experimental models.

The MTT method is an assay that determines cell viability *via* cell mitochondrial dehydrogenases. In our experiments, NaSe reduced cell viability by blocking intracellular mitochondrial pathways or exerting a direct toxic effect on cells at concentrations above 1 µM.

In our study, we observed that low doses of NaSe (<1 µM) exhibited ROS-scavenging properties. The identification of novel antioxidant compounds and the repurposing of known agents like NaSe remain highly relevant in current scientific research. There are many studies in the literature examining the beneficial effect of NaSe on ROS scavenging properties [20-23], and this feature is increasingly recognized by researchers. Given that fully understanding the mechanisms of drug candidates takes time, continued investigation into molecules like NaSe remains essential. At concentrations between 0.01-1 µM, NaSe may reduce intracellular ROS by enhancing the activity of antioxidant selenoproteins (*e.g.*, glutathione peroxidase, thioredoxin reductase), which neutralize ROS and maintain redox balance. This can prevent oxidative stress-induced apoptosis and support key osteogenic pathways like Nrf2/Keap1 and Wnt/β-catenin, which are crucial for osteoblast survival, differentiation, and mineralization. By maintaining optimal ROS levels, NaSe may foster a pro-osteogenic environment, promoting bone formation while preventing oxidative damage.

Bone formation and destruction remain in balance, but in some pathological conditions or malnutrition, this balance can be disrupted. When bone formation is inadequate, bones begin to soften and become porous, which causes bone fractures. There are many factors that affect bone formation, and selenium is one of these factors [24, 25]. It has been shown in many preclinical and clinical studies that selenium has beneficial effects on bone metabolism [9, 10, 26-32]. However, despite the beneficial effects of selenium, it has also been shown that it does not affect bone metabolism [33]. Investigating the effects of selenium on bone metabolism is very important to clarify this issue. We previously investigated the effects of selenomethionine on hFOB cells. When we compared this study with the results of our previous study, it was determined that both molecules had osteoblastic activity, but their concentration effects were different. The concentration of 15 μm demonstrated maximum efficacy for selenomethionine, while 0.1 μm emerged as the most effective concentration for NaSe. While the ALP level in NaSe increased by 136% at 0.1 μm, selenomethionine could reach these levels at 15 μm [34]. This difference is likely due to variations in selenium metabolism, cellular uptake, and redox activity. NaSe may rapidly be bioavailable and directly influences osteogenic pathways, while selenomethionine undergoes slower metabolic conversion. These findings highlight the distinct mechanisms by which selenium compounds regulate bone formation and suggest that NaSe may be more potent at lower doses.

We anticipate that these two studies will further promote the investigation into the effects of selenium derivatives on bone metabolism. The limitation of these two studies is that they have not been tested in experimental animals. Additionally, the long-term effects of NaSe could have been explored in this study, providing deeper insights into its sustained impact on osteoblastic activity. Furthermore, expanding the dose range would have allowed for a more comprehensive assessment of its toxicity at higher concentrations, offering a broader perspective on its safety profile. Sharma *et al.* tested NaSe on mouse pre-osteoblast MC3T3-E1 cells, and it was shown that NaSe increased osteogenic activity in these cells through the WNT signaling pathway [30].

Both our study and that of Sharma *et al.* demonstrated that NaSe has similar effects on osteoblasts across different species. However, notable differences were observed in the effective dose: while we identified 0.1 µM as the most effective concentration in human osteoblasts, Sharma *et al.* reported 3.2 µM as optimal in mouse osteoblast cells.

The dose differences from the two studies indicate that human osteoblast cells are more sensitive to NaSe than mouse osteoblasts. Even though animal and human cells are similar, they can sometimes produce different results under different conditions [35]. The superior effectiveness of 0.1 µM NaSe in enhancing mineralization may be attributed to its optimal balance between antioxidative protection and osteogenic stimulation. At this concentration, NaSe likely reduces excessive oxidative stress while supporting key signaling pathways (*e.g.*, Wnt/β-catenin, BMP-2/Smad) that promote osteoblast differentiation and mineralization. Higher doses may induce cytotoxic effects, disrupt mineral homeostasis, or interfere with selenoprotein function, leading to diminished mineralization.

## CONCLUSION

This study demonstrated that NaSe exhibits antioxidative and osteoblastic activity in hFOB cells, providing valuable insights into its potential role in bone metabolism. The determination of effective NaSe doses for cell viability, cytotoxicity, ROS scavenging, and osteoblastic activity contributes to the growing body of literature on the biological effects of selenium.

Nevertheless, despite these findings, significant gaps remain in our understanding of selenium’s precise role in bone health-particularly regarding its long-term effects, underlying molecular mechanisms, and interactions with other essential trace elements. Future research should prioritize animal and clinical studies to validate these *in vitro* findings and explore the therapeutic potential of NaSe in bone-related disorders. Investigating the bioavailability, safety, and optimal dosing of selenium compounds, as well as their interplay with key minerals in bone metabolism, will be crucial for translating these findings into clinical applications. Expanding our knowledge in these areas may help establish selenium-based strategies for bone disease prevention and treatment.

## Data Availability

All the data and supporting information are provided within the article.

## LIST OF ABBREVIATIONS

LDH: Lactate Dehydrogenase
ALP: Alkaline Phosphatase
ROS: Reactive Oxygen Species
AR: Alizarin Red
